# Case Series of Brittle Cornea Syndrome

**DOI:** 10.1155/2020/4381273

**Published:** 2020-03-20

**Authors:** Taher Eleiwa, Mariam Raheem, Nimesh A. Patel, Audina M. Berrocal, Alana Grajewski, Mohamed Abou Shousha

**Affiliations:** ^1^Bascom Palmer Eye Institute, Miller School of Medicine, University of Miami, Miami, FL, USA; ^2^Department of Ophthalmology, Faculty of Medicine, Benha University, Benha, Egypt

## Abstract

**Purpose:**

This case series demonstrate diagnostic features, treatment options, and challenges for Brittle Cornea Syndrome. *Observations*. Three cases presented with bluish sclera and extremely thin cornea. Genetic workup was performed and confirmed the diagnosis of Brittle Cornea Syndrome, a rare autosomal recessive disorder characterized by corneal thinning and blue sclera. Case 1 was a 4-year-old boy who developed cataract and glaucoma after undergoing right tectonic penetrating keratoplasty (PK) secondary to a spontaneous corneal rupture. Glaucoma was controlled medically. Later, the kid underwent right transcorneal lensectomy and vitrectomy with synechiolysis. After 6 weeks, he sustained graft dehiscence that was repaired using onlay patch graft. Case 2 was a 7-year-old boy who underwent PK in the right eye, then a pericardial patch graft in the left eye following spontaneous corneal rupture. Glaucoma in both eyes was controlled medically. Case 3 was the 2-year-old sister of the 2^nd^ case. She had a pachymetry of 238 *μ*m OD and 254 *μ*m OD and 254

**Conclusions:**

Long-term follow-up of children diagnosed with Brittle Cornea Syndrome is paramount to minimize the morbidity of corneal rupture and late-onset extraocular conditions.

## 1. Introduction

Brittle Cornea Syndrome is an autosomal recessive disorder that is characterized by corneal thinning and blue sclera [1]. This places the patient at an increased risk of spontaneous rupture or rupture due to minor trauma. Due to the severity of the corneoscleral thinning, surgical repair and postoperative management pose extra challenges. Here, we present clinical outcomes of 3 cases with Brittle Cornea Syndrome.

## 2. Findings

### 2.1. Case 1

A 4-year-old Indian boy was brought by his parents to Bascom Palmer Eye Institute three months after having a penetrating keratoplasty (PK) done in his right eye. Three months earlier, the patient had rubbed his right eye while eating. Initially, the patient was seen by his pediatrician and was started on topical antibiotics. The patient returned with white discharge from the right eye. The patient was referred to an ophthalmologist and immediately sent him to the Emergency Room where he was admitted and had a PK for right perforated cornea.

**Figure 1:**
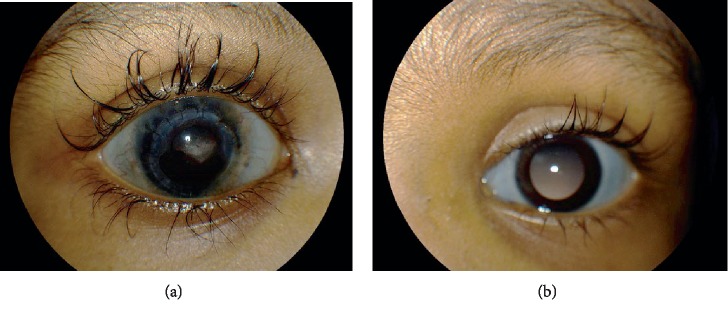
Slit-lamp photography showing bilateral blue sclera in the right (a) and left (b) eye. Corneal graft in the right eye (1a) is large and clear with intact sutures.

Upon initial examination, the patient had bluish sclera bilaterally (Figure 1). The patient's visual acuity was light perception in the right eye and fix and follow in the left eye. The graft was large and clear (Figure 1(a)). The patient had history of glaucoma in the right eye that was treated medically.

**Figure 2:**
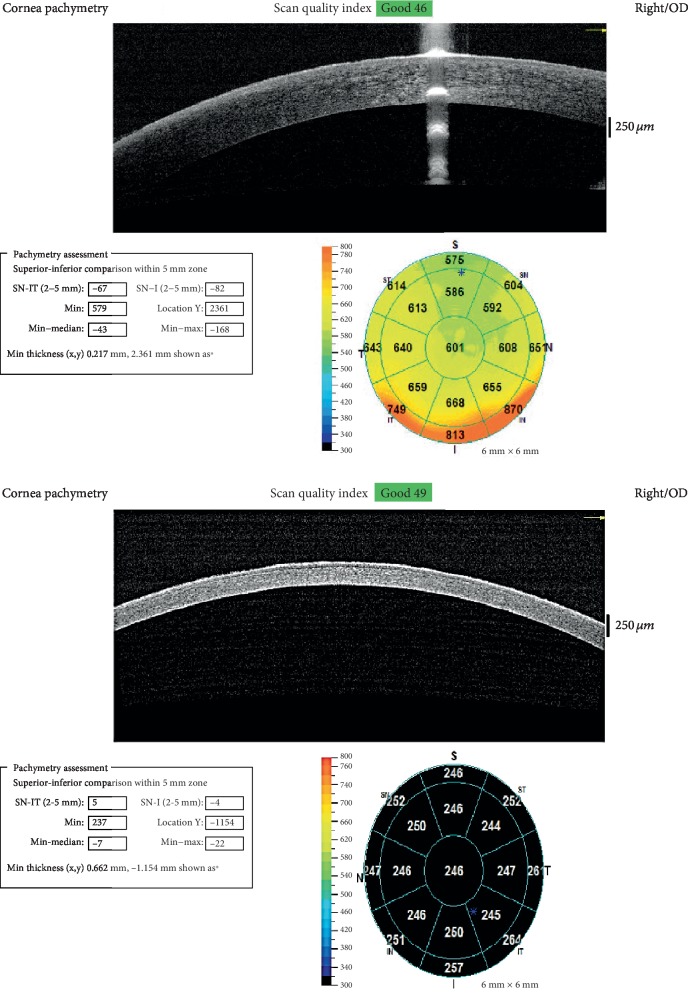
Anterior segment OCT showing corneal thickness maps of both eyes. The right cornea graft has a minimal thickness of 579 *μ*m versus 237 *μ*m in the left eye with Brittle Cornea.

Examination under anesthesia revealed that the patient's IOP in the right eye was 34 mmHg and 14 mmHg in the left eye. The Retinoscopy in the right eye revealed −6.00 + 3.00 × 180 and −3.00 + 1.00 × 90 in the left eye. In the right eye, the corneal diameters were 11.5 mm horizontally and 12.0 mm vertically. In the left eye, the corneal diameters were 11.0 mm horizontally and 11.0 mm vertically. The anterior segment exam of the right eye showed peripheral anterior synechiae, intact corneal graft sutures, and no signs of graft rejection. Anterior segment exam of the left eye revealed clear, thin cornea, and optically empty anterior chamber. The cup/disc ratio of the right eye was 0.9 and 0.5 in the left eye. B-scan revealed a posterior staphyloma in the left eye. The axial length was 25.5 mm in the right eye and 23.7 mm in the left eye. An anterior segment OCT was obtained while patient was under anesthesia (Figure 2). The thinnest point of the left cornea determined by the OCT was 237 *μ*m.

There was a concern for Osteogenesis Imperfecta (type I) indicated by thin corneas and bilateral bluish sclera. The patient was sent for genetic analysis. The geneticist noted that the mother and father were first cousins and both had bluish sclera, but were otherwise healthy. Physical findings included dolichocephaly, a prominent forehead, and increased joint flexibility. A buccal sample was collected and tested for 49 different genes, including the ZNF469. The ZNF469 gene showed a homozygous mutation from the patient's buccal samples, confirming the diagnosis of Brittle Cornea Syndrome.

**Figure 3:**
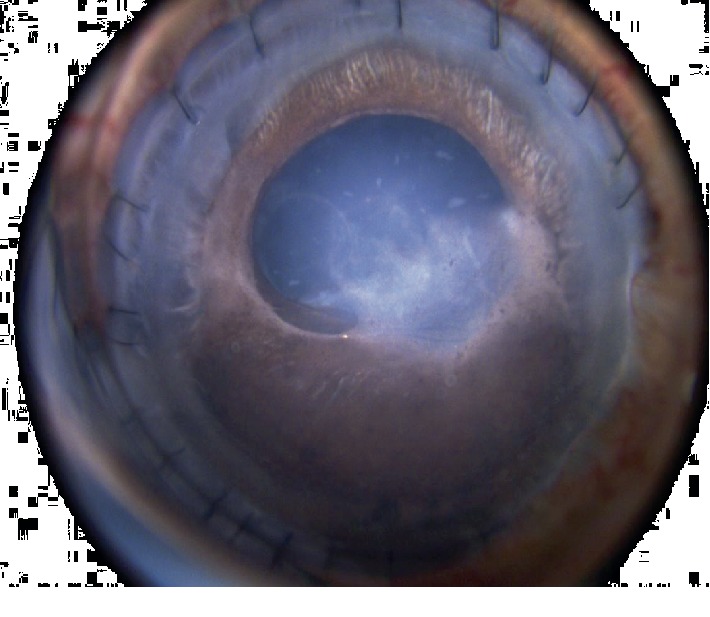
Demonstration of cataract and inferior posterior synechiae of the right eye. The corneal graft is clear with intact sutures.

**Figure 4:**
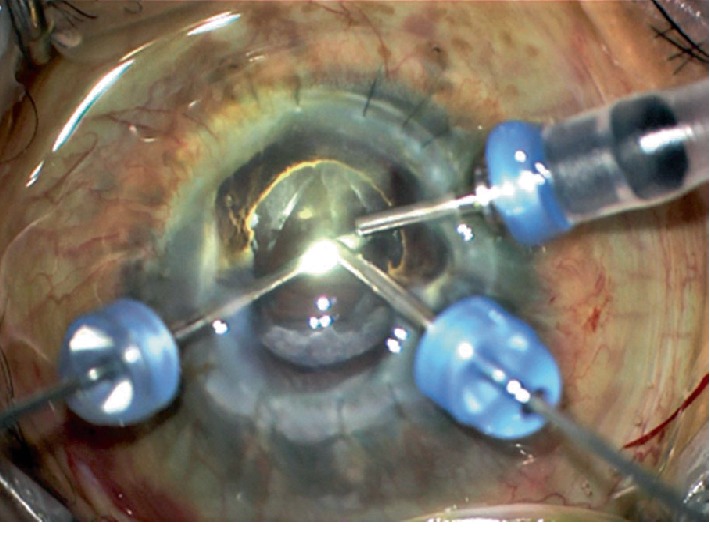
Screenshot captured during the cataract removal surgery demonstrating the transcorneal placement of the trocars 0.5 mm anterior to the graft-host junction.

**Figure 5:**
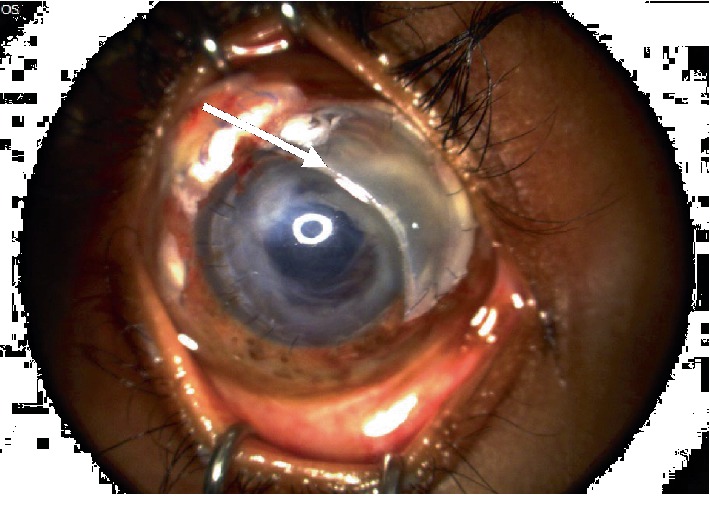
Slit-lamp photography of the right eye showing a superonasal corneal patch graft (arrow).

On follow up, the patient was found to have a secondary cataract in his right eye with inferior posterior synechiae and a fibrotic capsule adhered to the iris. The corneal graft was clear with intact sutures (Figure 3). Two months later, the patient underwent a transcorneal lensectomy (through the donor cornea), limited vitrectomy without lifting the hyaloid, synechiolysis, and removal of iris membrane in the right eye (Figure 4, supplementary video ([Supplementary-material supplementary-material-1])). This was indicated because the cataract was visually significant and presented difficulty when trying to examine the fundus. One day after surgery, the VA was counting fingers and improved to 20/200 2 weeks later. The VA in the left eye was 20/80 without correction. Patient's IOP is well controlled on medications. Unfortunately, 6 weeks after the procedure, the patient presented with a 3-clock hour dehiscence (from the area of the original cornea) of the corneal graft superonasally with no uveal prolapse. The patient underwent repair with a corneal patch graft (Figure 5).

### 2.2. Case 2 and 3: A Case of Two Siblings of Consanguineous Parents

**Figure 6:**
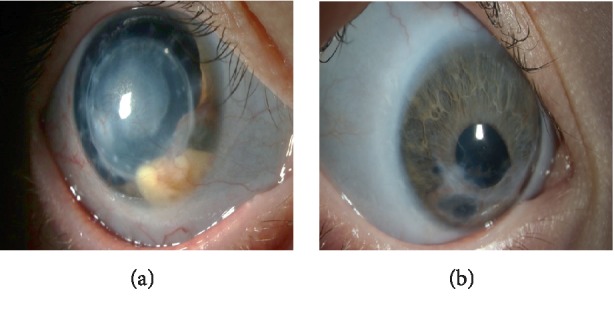
Slit-lamp photography showing vascularized corneal graft with inferonasal secondary lipid keratopathy in the right eye (a) and inferior vascularized leukoma adherent in the left eye.

**Figure 7:**
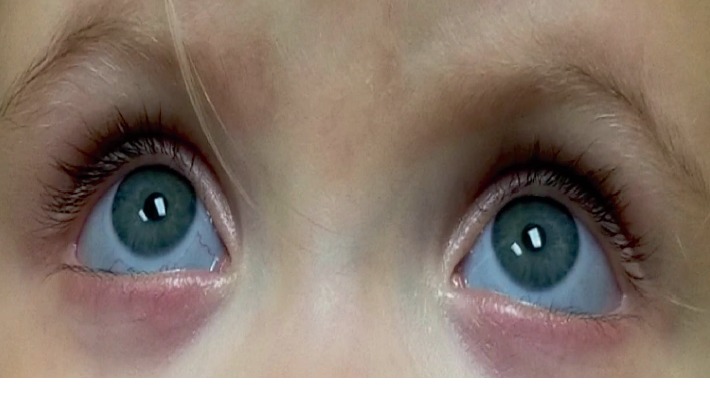
External photography showing blue sclera on a 2-year-old girl with Brittle Cornea Syndrome.

A 7-year-old boy presented with parents and his 2-year-old sister for evaluation of Brittle Cornea Syndrome, which was confirmed by genetic testing and presented for a second opinion. The 7-year-old boy underwent a PK following spontaneous rupture of the right cornea when the patient was 4 years old. One year later, he underwent a pericardial patch graft following a spontaneous rupture of the left eye which has since fallen and scarred. On examination, the patient had a corneal graft which was vascularized and had inferonasal secondary lipid keratopathy in the right eye (Figure 6). In the left eye, patient's superior cornea was clear, and there was an inferior vascularized leukoma adherent (Figure 6). The patient's 2-year-old sister had no previous surgeries. Both patients had bluish sclera bilaterally (Figure 7).  Table 1 summarizes the clinical characteristics in both patients. The patient's parents were instructed to take protective measures for both children and to continue with follow-up visits. Parents were also instructed to have regular screenings for hearing loss, dental abnormalities, and bone deformities during development.

## 3. Discussion

Brittle Cornea Syndrome is an autosomal recessive syndrome that affects connective tissues. Type I is diagnosed through the identification of mutation in the ZNF469 gene which encodes the transcription regulator that participate in pathways regulating extracellular matrix and collagen synthesis [2]. This results in severe thinning of the cornea and prevents proper development of the anterior chamber and creates a high risk of spontaneous corneal rupture [3]. Type II results from a mutation of PRDM 5. The exact phenotype of the type II mutation is unclear.

Spontaneous rupture or rupture following a minor trauma is a significant risk in patients with Brittle Cornea Syndrome. At glance, there can be a concern for potential child abuse. In our patients, there was no evidence of child abuse. Parents were compliant with all medical instructions as well as office visits and were attentive about their children's progress.

In this series, two out of three cases had corneal perforations. In patients diagnosed with Brittle Cornea Syndrome, precautions are taken in order to prevent ruptures such as polycarbonate goggles and close monitoring of symptoms [4]. Wound repair after rupture can result in further tissue loss. Additionally, sutures that are implanted during a primary keratoplasty have an increased risk of cheese-wiring due to the thin cornea, and there is a risk of overriding due to mismatch between graft and host thickness, both of which could result in increased risk of postoperative infection and leakage [5].

The impaired collagen integrity with Brittle Cornea Syndrome provided a challenge in the surgical approach and incision placement for the lensectomy and vitrectomy in case 1. On inspection under anesthesia, there was severe host circumferential limbal and scleral thinning. The concern with utilizing conventional limbal wounds was the possible inability to reapproximate the compromised tissues [5]. The approach taken was to place the trocars through the donor keratoplasty tissue, 0.5 mm anterior to the graft-host junction (Supplementary video). This allowed for adequate access for lensectomy, anterior chamber stability, and uncomplicated closure. To the authors' knowledge, there are no prior reports on lensectomy or the use of trocar placement through donor tissue in brittle cornea syndrome. There was dehiscence of the graft 6 weeks later. It is possible that surgical manipulation further wreaked the zone of attachment. Again, the integrity of the tissue provided a challenge for repair and a patch graft was needed. The choroidals resolved and the retina remained attached. The problem is that the original cornea continues to suffer from progressive weakness.

The issues with potential wound leak add to the well-established risks of keratoplasty in pediatric patients. Pediatric patients have a greater risk of postoperative inflammatory reaction and increased fibrin deposition [6]. 20-50% of patients under 5 years old have graft failure attributed to the increased inflammatory activity of patients in this age group [7]. Overall, patients who receive grafts under 4 years of age have a 65% survival rate in the first year [8]. Patients aged 5-12 have an increased chance of graft survival [9]. Postoperative steroid should be used cautiously in patients with brittle cornea syndrome due to the negative effects of steroids on collagen synthesis as well as the risk of secondary glaucoma development [10]. The management of comorbidities also becomes complicated by the graft. About 56% of grafts in pediatric eyes that underwent removal of cataract, IOL implantation, or YAG laser capsulotomy had failed [9].

In some cases of Brittle Cornea Syndrome, there is progressive corneal thinning which may result in the development of keratoglobus or keratoconus. As a result, corneal cross-linking using a modified method in order to account for the thin cornea has been tried as a treatment in pediatric patients [11]. It is worth mentioning that corneal perforations after corneal cross-linking for keratoconus in a woman harboring potentially pathogenic variants in the ZNF469 gene have been recently reported [12]. Additionally, in a case of a monocular patient due to previous rupture and severe corneal ectasia in the seeing eye may have an onlay corneoscleral graft [13]. Five out of six corneas that had surgery for tectonic support placement had successful visual improvement [14].

Glaucoma is the leading cause of irreversible blindness postkeratoplasty [15] and is a leading cause of graft failure [10]. Glaucoma management in pediatric patients is challenging due to the lack of cooperation of these patients. However, effective management is necessary in order to prevent visual loss and prevent damage to the cornea and optic nerve [16]. Also, glaucoma after corneal graft surgery can potentially lead to visual loss and graft failure [17]. The risk of developing glaucoma after a penetrating keratoplasty ranges from 5.5-31% in the early postoperative period and can be as high as 17-35% in the late postoperative period [18]. Patients with peripheral anterior synechiae are at high risk for developing glaucoma [18]. Additionally, in a retrospective study on patients with fragile corneas, it was found that 50% of pediatric patients developed glaucoma after penetrating keratoplasty [19]. In the meantime, it has been credibly proven that corneal thickness exerts an influence on the accuracy of applanation tonometry by about 1 mmHg per 25 microns corneal thickness change [20]. Therefore, IOP measurements have to be corrected in the setting of Brittle Cornea Syndrome.

The decision making for glaucoma management with the thinning present is complex. The absence of reliable sclera poses difficulties in suturing of a plate for a glaucoma drainage implant. An option could be posterior plate location and a pars plana location of the tube at the time of lensectomy and vitrectomy. Also, the use of fibrin glue to secure the plate to the sclera as a substitute for the sutures has been reported [21–23]. Another approach, if topical pharmacotherapy is not successful, is cyclophotocoagulation with adjusted energy parameters [24]. This could again be achieved transsclerally or with an endoscopic technique at the time of the lensectomy.

## 4. Conclusion

Patients with Brittle Cornea Syndrome require close monitoring, use of protective polycarbonate lenses, and appropriate lifestyle modifications in order to prevent ocular rupture. High-risk patients may require prophylactic corneal cross-linking or corneoscleral grafts. Surgical management in these cases can provide unique challenges due to impaired wound healing. Early diagnosis and continuous care with a multidisciplinary approach, including glaucoma management, are essential in order to prevent vision loss.

## Figures and Tables

**Table 1:** Clinical features of case 2 and case 3.

	Case 2	Case 3
Age	7 years	2 years
Gender	Male	Female
Gene	ZNF469	ZNF469
Presenting visual acuity	OD: counting fingers OS: 20/70 cc	OD: 20/70 cc OS: 20/60 cc
IOP	OD: 16 mmHg OS: 15 mmHg	OD: 16 mmHg OS: 14 mmHg
Minimal corneal thickness	OD: 366 *μ*m OS: 232 *μ*m	OD: 238 *μ*m OS: 254 *μ*m
Refraction	OD: +12.00 OS: −14.00/+4.75 × 085	OD: −9.25/+4.25 × 090 OS: −9.25/+4.50 × 090
Surgical history	OD: penetrating keratoplasty OS: pericardial patch graft	None
Medications	OU: timolol 0.5% bid	OU: timolol 0.5% bid

## Supplementary Materials

Supplementary Materials Supplementary video: showing the approach of transcorneal lensectomy with synechiolysis and core vitrectomy in the right eye of case 1. The trocars were placed 0.5 mm anterior to the graft-host junction due to severe host circumferential corneoscleral thinning.
